# Anal HPV shedding assessed by self-sampling and multiplex real-time PCR among men who have sex with men in N’Djamena, Chad: a feasibility and acceptability study

**DOI:** 10.1371/journal.pone.0340799

**Published:** 2026-01-14

**Authors:** Donato Koyalta, Zita Aleyo Nodjikouambaye, Jonathan Muwonga Tukisadila, Hachim Djamal Abdoulaye Bargo, Suitombaye Noubaramadji Yamti, Amine Akouya, Ralph-Sydney Mboumba Bouassa, Laurent Belec

**Affiliations:** 1 Université de N’Djamena, N’Djamena, Chad; 2 Ecole Doctorale Régionale d’Infectiologie Tropicale de Franceville, Franceville, Gabon; 3 Laboratoire de Biosûreté et des Epidémies (LaBiEp), N’Djamena, Chad; 4 Laboratoire de Virologie, Hôpital Européen Georges Pompidou, Paris, France; 5 Laboratoire des Grandes Epidemies Tropicales “LAGET”, CHU le Bon Samaritain, N’Djamena, Chad; 6 Department of Family Medicine, Faculty of Medicine, Institut du Savoir Montfort, Montfort Hospital, University of Ottawa, Ottawa, Canada; 7 Faculté de Médecine Paris Descartes, Université Paris Cité, Paris, France; Cairo University Faculty of Veterinary Medicine, EGYPT

## Abstract

**Background:**

High-risk (HR) human papillomavirus (HPV) infection remains a great concern in sub-Saharan Africa in men who have sex with men (MSM). The prevalence of anal shedding of HPV and associated risk factors was estimated for the first time in a cross-sectional observational study covering MSM living in N’Djamena, the capital city of Chad.

**Methods:**

MSM were recruited from the community in 21 sites in neighborhoods of 5 districts randomly selected in N’Djamena by respondent-driven sampling (RDS) method. Anal Collector V-Veil UP2™ device was used for anal canal self-sampling. Manual silica-extracted DNA was subjected for HPV detection and genotyping using BMRT Human Papillomavirus Genotyping Real Time PCR assay (Jiangsu Bioperfectus Technologies Co., Ltd., Taizhou, China). HIV serostatus was assessed using two rapid tests in series.

**Results:**

A total of 70 MSM (mean age: 29.9 years; range, 18–50) were included. The overall acceptability to practice veil-based anal self-sampling was 95.9%. The usability of the veil collector device was high (92.3%), with easy understandable instructions for use and correct placement in the anal canal. Satisfaction questionnaire reported high overall feeling, intimacy respect and lack of shame. The majority of MSM (44/70, 62.8%) showed anal shedding of HPV DNA, with HR-HPV frequently detected (38,70, 54.3%), including HPV-33 (30/70, 42.9%) HPV-68 (16/70, 22.9%), HPV-18 (4/70, 5.7%), HPV-35 (3/70, 4.3%), HPV-58 (2/70, 2.9%), and HPV-45 (1/70, 1.4%). The distribution of genotypes in HR-HPV DNA-positive MSM revealed that HPV-33 (30/70; 42.9%) was the predominant genotype, followed by the HPV-68 (16/70; 22.9%), HPV-18 (4/70; 5.7%), HPV-35 (3/70; 4.3%), HPV-58 (2/70; 2.9%), and HPV-45, HPV-51 and HPV-56 (each type, 1/70;1.4%).

Among all HPV detected, only 42 HPV (36.8%) were covered by Gardasil-9^®^ vaccine, including the HR-HPV-33, −18, −58 and −45, and the low risk-HPV-6 (5.7%) and HPV-11 (1.4%). The majority of detected HPV were non-covered by Gardasil-9^®^ vaccine (63.1%). Overall HIV prevalence was 5.7%.

**Conclusions:**

Taken together, these observations point the MSM population in N’Djamena as a very particular core group of HIV and HPV transmission. HIV prevalence was higher than that of general adult population, but limited to only one MSM of twenty. The RDS method of recruitment allowed to include MSM likely belonging to the same sexual network of HPV transmission leading to the selection of an atypical and specific profile of anal HPV distribution. The potential efficacy of HPV prophylactic vaccination in this population can be estimated at relatively weak.

## Introduction

Men in sub-Saharan Africa (SSA) are a significant reservoir for human papillomavirus (HPV) infection, with regional prevalence consistently above 25% [1,2]. A recent review found the highest HPV prevalence in SSA men [excluding men who have sex with men (MSM)] at 37% [3]. This underscores the importance of including men in HPV control efforts.

MSM in SSA face a high burden of sexually transmitted infections (STIs), particularly anogenital HPV, which causes warts and cancers of the anus, penis, and oropharynx [4–10]. Anal high-risk HPV (HR-HPV) poses a growing threat, as MSM are 20 times more likely than heterosexual men to develop HR-HPV-associated anal cancer [11,12]. This is compounded by social stigma and the criminalization of male homosexuality, which limit epidemiological research [13,14]. Despite this, MSM are recognized as a key vulnerable population for HIV infection [15], an independent risk factor for anal HR-HPV [16,17]. The high co-prevalence of anal HR-HPV and HIV suggests a significant risk for HPV-associated anal cancer, though comprehensive data is scarce [9,18–22].

A wide diversity of predominant HPV genotypes has been observed across African countries, suggesting unique regional variations in HPV epidemiology [9,15,19,23]. For example, while HPV-16 was predominant in MSM in Mali and South Africa [15,23], non-vaccine HPV-35 was prevalent in Nigeria and the Central African Republic [9,19]. These findings highlight MSM in SSA as a high-risk group with genotype distributions potentially distinct from global trends [24]. To implement effective prevention, especially with prophylactic HPV vaccines, establishing the molecular distribution of circulating predominant HPV genotypes in African MSM is essential.

Chad, a large, landlocked country in West and Central Africa, has an adult HIV prevalence of 1.0% [25]. HPV infection has recently been documented in Chadian women, revealing a high burden of cervical HR-HPV infection (15.8%) [26]. Primary prevention through prophylactic vaccination could be a vital option for MSM in Chad, who likely lack access to diagnostic exams and care. However, no epidemiological data is available on the distribution of circulating HR-HPV genotypes in Chadian MSM to our knowledge.

Given that HPV epidemiology in MSM can be area-specific, with implications for vaccine efficacy, we designed this cross-sectional study. Our aims are to assess the prevalence and type distribution of anal HPV infection and associated risk factors in a population of MSM living in N’Djamena, the capital city of Chad. Additionally, for the first time in an African MSM population, to the best of our knowledge, we evaluated the acceptability, usability, and satisfaction of anal self-sampling [27–29].

## Materials and methods

**Study design and population.** The *ANAUTO-CHAD* study was a descriptive, quantitative, population-based, cross-sectional survey, using a face-to-face questionnaire to collect data and the Veil Collector V-Veil UP2™ device (V-Veil-Up Production SRL, Pitesti, Romania) for the characterization of anal HPV infection by anal self-sampling among adult MSM living in N’Djamena, Chad, recruited from the community. Standards for Reporting of Diagnostic Accuracy (STARD) guidelines were used for reporting the study [30,31].

From the 1^st^ of August to the 31^st^ of September 2023, Chadian MSM were approached and asked to participate to the study by peer educators from the Chadian National Network of Associations of People Living with HIV [so-called *Réseau National Tchadien des Associations des Personnes vivant avec le VIH (RNTAP+*)], N’Djamena, Chad. The *RNTAP*+ is a specialized non-governmental organization (NGO) exclusively dedicated to key populations. It offers counseling, testing, care, and support to MSM from N’Djamena. MSM regularly attend the center for HIV and STIs screening and care, to receive specific treatment, HIV counseling and HIV global support for those tested positive. The diagnosis of STIs symptoms was both clinical and anamnestic, and was performed by a healthcare provider. All participants volunteered to take part in the study.

For purposes of the study, a specific strategy involving peer educators from the NGO *RNTAP+* was adopted in order to confirm the accuracy of homosexuality of included MSM. Thus, the main inclusion criteria were being adult (≥18 years) and approved by his peers as having sex with men. The possibility of being followed up for at least 3 months was also requested at inclusion, and the participants provided a fully informed medical and socio-demographic record, and to sign the informed consent form. MSM who did not meet the inclusion criteria and those who had condomless receptive anal intercourse less than 24 hours before the initiation visit for anal sampling were excluded from the study. Furthermore, at the initiation visit, an additional 4 hours period after defecation was set up before carrying out the anal sampling, to avoid contamination of the anal swab with fecal residues which could impair PCR efficiency. An interview using the same face-to-face standardized questionnaire was conducted at inclusion to collect socio-demographic characteristics, behavioral data and medical information on HIV and STIs.

The capital city N’Djamena comprises 10 districts, which include a variable number of neighborhoods. Twenty-one sites in neighborhoods of 5 districts randomly selected out of 10 were further chosen for study inclusion [1^st^ district (n=1): Farcha; 3^rd^ district (n=3): Ardep Djoumal, Kabalaye, Sabangali; 4^th^ district (n=1): Am-Riguebe; 6^th^ district (n=2): Moursal, Paris-Congo; 7^th^ district (n=9): Ambata, Amtoukoui, Atrone, Boutalbagara, Chagoua, Dembé, Gassi, Habena, Kilwiti; 9^th^ district (n=5): Dingangali, Gardolé Djidid, Ngueli, Tourkra, Walia].

The study used the respondent-driven sampling (RDS) method to deliberately select five initial MSM participants (referred as “seeds”) that were purposively selected by the investigators as they were well-connected with MSM networks in N’Djamena, to serve as initial contacts for chain recruitment of MSM through their network, as previously described [32]. All seeds were given three coupons and asked to recruit three MSM from among their peers. Thus, the seeds invited other MSM to participate to the study and to be interviewed. Recruited MSM eligible and consenting to be seeds, became themselves recruiters. Recruits were encouraged to take part in the study, without being paid, and to recruit other MSM of their network. This process was carried out in three waves of recruitment (with wave zero being defined as the seeds, wave one as their recruits, etc). Due to the absence of any previous knowledge on the prevalence of anal HPV infections among MSM in Chad, no pre-determined sample size was fixed.

**Fig 1:**
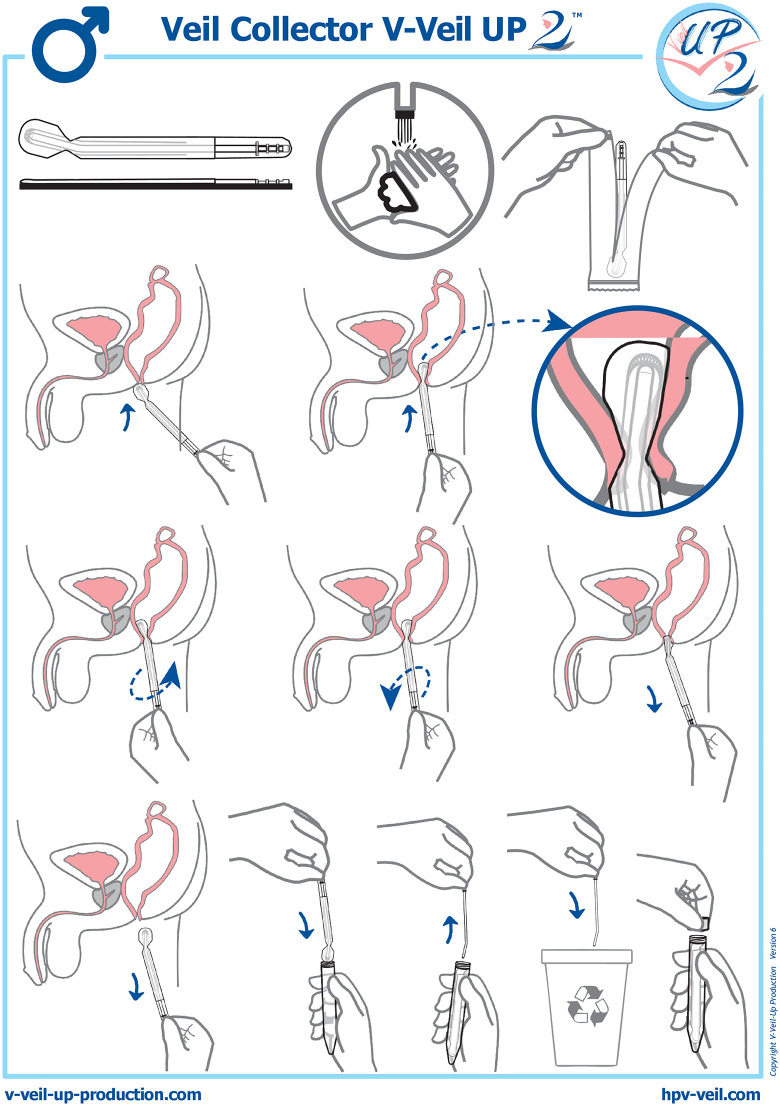
Successive steps of the instructions for use of Veil Collector V-Veil UP2™ (V-Veil-Up Production SRL, Romania; https://www.v-veil-up-production.com/). The Veil Collector V-Veil UP2™ device is intended for the safe and non-invasive collection of anal secretions from the anal canal. The device is conceived for self-sampling collection in privacy, and collected anal canal secretions may be further gathered from the veil to be used for biological analysis. The Veil Collector V-Veil UP2™ consists in a pocket in non-woven hydrophilic polyethylene, a V-shaped end and a plastic applicator, whose nozzle is curved, which allows the veil to better fit the canal anal. The plastic applicator makes possible to push the veil to the contact of the anal verge after its intrusion into the pocket. The veil is applied 1-2 cm from the anal canal using the plastic applicator. The veil is rotated 1-2 times for self-collection, then removed. By pulling on the V-shaped end of the veil, the applicator can be easily removed. The impregnated veil is placed into a 15 mL plastic box containing 10 mL of PBS to prevent drying of the sample, and is closed with his cap. By its conception, the device does not absorb the liquids and allowed full recovery of collected biological material. The veil must be stored in a clean, dry place away from heat sources. Reprinted from V-Veil-Up Production SRL, Romania, under a CC BY license, with permission from Bernard Chaffringeon, original copyright 2024, version 6.

**Procedure and sampling collection.** In each inclusion site, the seeds contacted adult MSM in hot spots, community churches and mosques or MSM networks during a one-month period and proposed them to be included in the *ANAUTO-CHAD* study after an oral explanation on the objectives of the survey, mainly focused on sensitization on anal cancer and prevention strategies against anal HPV acquisition. After written consent, the selected MSM were invited, with paid transportation, to come to health centers involving medical staff from the NGO *RNTAP+* to use themselves the anal veil for self-sampling in a room allowing privacy, or to benefit from an anal swab sample taken by a healthcare professional in the event of refusal the anal self-sampling. The study participant accepting anal self-sampling received from a nurse a 15-minutes training on how to use the Veil Collector V-Veil UP2™ device for anal self-sampling (Fig 1). After instructing the participant, the nurse left the sampling room and the participant then performed himself the self-sampling, without any help from the nurse. The participant followed the instructions for use of the V Veil Collector V-Veil UP2™ device (Fig 1). Immediately after removal, the impregnated veil was placed into a 15 mL plastic box containing 10 mL of phosphate-buffered saline (PBS) solution to prevent the drying of the sample, and the box was closed. The nurse verified that the PBS buffer completely submerged the veil and checked that the identification number (assigned study code) in the label on the collection box corresponded effectively to the participant. The veil filled of secretions in its box was then placed in the cold (ice packs or crushed ice), and was sent to the virology laboratory of the university *Hôpital Général de Référence Nationale* (HGRN), N’Djamena, Chad, within 4 hours, for storage at −80°C before the DNA extraction procedure.

After returning the veil specimen, a second nurse administered acceptability, usability and satisfaction questionnaires on the participant’s experiences about the veil-based anal self-collection. The objectives of the acceptability questionnaire were to evaluate the study MSM’s experiences related to the perception of care, comfort, privacy, embarrassment, or pain associated to the self-collection method. The satisfaction questionnaire consisted in questions regarding the ability to understand the instructions for use of the Collector V-Veil UP2™ device and its component and to perform self-collection in medical facilities setting or at home, and also assessed difficulties encountered during veil-collection.

**Samples processing and DNA extraction.** Serum samples were collected by venipuncture for serological testing of HIV infection, as recommended by the national algorithm of the Chad HIV National Control Program, using DETERMINE™ HIV-1/2 (Abbott, Chicago, Illinois, USA) for HIV screening and another available HIV test among those proposed by the Ministry of Health for confirmation [33,34].

Nucleic acid extraction was carried out at HGRN using QIAamp^®^ DNA Mini Kit (Qiagen, Hilden, Germany) according to manufacturer’s instructions and eluted in 100 µL of the kit elution buffer before genotyping. Extracted DNA was aliquoted and stored at –80°C until analysis. Frozen aliquots of extracted DNA were further sent in frozen ice packs in the *Laboratoire de Biosûreté et des Epidémies (LaBiEp),* N’Djamena, Chad, for HPV molecular detection.

**HPV detection and genotyping by multiplex real-time PCR.** HPV detection and genotyping was carried out using the fluorescence-based Bioperfectus Multiplex Real Time (BMRT) Human Papillomavirus Genotyping Real Time PCR Kit (Jiangsu Bioperfectus Technologies Co., Ltd., Taizhou, Jiangsu Province, China). The BMRT HPV kit contains primers and corresponding TaqMan probes that amplify a 100-base pairs L1 amplicon, as previously described [35]. According to the HPV classification nomenclature provided by the International Agency for Research on Cancer (IARC) [36], the BMRT HPV Genotyping Real Time PCR Kit allows to distinguish each of the 21 most prevalent HPV genotypes, including 13 HR-HPV (HPV-16, -18, -31, -33, -35, -39, -45, -51, -52, -56, -58, -59 and -68), 5 possibly oncogenic HPV (HPV-26, -53, -66, -73 and -82) and 3 LR-HPV (HPV-6, -11 and -81). The BMRT HPV kit was used according to manufacturer’s instructions, as described previously [37]. An internal control with the housekeeping single-copy TOP3 gene encoding human DNA topoisomerase III [38] was set to identify possible PCR inhibition and to confirm the reliability of the kit’s reagents. PCR was performed on the Mic Real-Time PCR System [Bio Molecular Systems (BMS), Upper Coomera, Australia].

**Statistical analysis**. Means and standard deviations (SD) were calculated for quantitative variables and proportions for categorical variables. The results were presented along with their 95% confidence interval (CI) using the Wilson score bounds for categorical variables [39]. The overall prevalence of HPV was assessed for any HPV genotypes, HR-genotypes and HPV genotypes targeted by the 4- and 9-valent Gardasil-9^®^ vaccines (Merck & Co. Inc., New Jersey, USA). The acceptability of the veil-based anal self-collection was assessed using an arbitrary quantitative Likert scale [40] based on four different scale ranging from 1 (most difficult), 2 (difficult), 3 (easy) to 4 (= very easy or comfortable). Similarly, the practicability and satisfaction regarding the veil self-collection method was assessed using another arbitrary quantitative Likert scale based on four different scale ranging from 1 (less favorable), 2 (moderately favorable), 3 (favorable) to 4 (= most favorable). The mean and standard deviation for Likert scale data were calculated for each acceptability and satisfaction item of the face-to-face questionnaires. A two-tailed P-value less than 0.05 was considered statistically significant. MedCalc^®^ version 23.0.9 (MedCalc Sofware Ltd, Ostend, Belgium) was used for all statistical analyses.

**Ethics statement.** The study was conducted in compliance with the ethical standards of the responsible institution on human subjects as well as with the Helsinki Declaration. The approval of the study was obtained from national ethics committee so-called *Comité National de Bioéthique du Tchad* (048/PT/PMT/MESRIS/SE/SG/CNBT/SG/2023). All participants were adults (18 years and older) and signed the informed consent form prior completing the study questionnaire. The authors did not have access to identifiable participant information during or after data collection.

## Results

**Characteristics of study population.** A total of 75 MSM from the 21 inclusion sites accepted to participate to the study. Five MSM were excluded, including 3 who refused anal-self sampling and 2 who had unprotected anal sex for less than 24 hours. Finally, a total of 70 MSM (mean age, 37.5 years; range, 18–50) were included in the study by RDS. Their socio-demographic characteristics, HIV serostatus, past history of STIs, and sexual behavior, are summarized in the Table 1.

**Table 1:** Baseline characteristics and anal detection of HPV by the BMRT Human Papillomavirus Genotyping Real Time PCR Kit (Jiangsu Bioperfectus Technologies Co., Ltd.) among the 70 study men who have sex with men (MSM) living in N’Djamena, Chad.

Characteristics	Overall
**Number**	70
**Age in years [*mean (range)*]**	29.9 (18-50)
**Education [*n (%)*]**	
Never been to school	16 (22.6)
Primary school	39 (55.7)
Secondary school	11 (15.7)
University	4 (6.0)
**Occupation [*n (%)*]**	
None	46 (65.7)
Employed or civil servant	13 (18.6)
Student	11 (15.7)
**Family situation [*n (%)*]**	
Single	62 (88.6)
Couple	8 (11.4)
**Age at first intercourse in years [*mean (range)*]**	15.9 (9-22)
**Number of sexual partners in last 6 months [*mean (range)*]**	6.15 (1-14)
**Number of sexual partners in last 6 months [*n (%)*]**	
1-5	39 (55.7)
5-10	19 (27.1)
> 10	12 (17.2)
**Multipartnership [*n (%)*]**	63 (90.0)
**Use of condom [*n (%)*]**	
Never	2 (2.8)
Rarely	8 (11.4)
Sometimes	56 (80.0)
Always	4 (5.8)
**Receptive anal intercourse [*n (%)*]**	58 (82.8)
**Insertive anal intercourse [*n (%)*]**	28 (40.0)
**Anal penetration with shared object [*n (%)*]**	14 (20.0)
**Receptive oral sex [*n (%)*]**	41 (58.6)
**Insertive oral sex [*n (%)*]**	32 (45.7)
**HIV infection [*n (%)*]**	4 (5.7)
**Under HAART [*n (%)*]**	3 (75.0)
**Genital STI syphilis [*n (%)*]**	1 (1.4)
**Genital STI *Herpes* [*n (%)*]**	5 (7.1)
**Anal lesions [*n (%)*]**	7 (10.0)
**Condyloma [*n (%)*]**	6 (8.6)
**Anal *Herpes* [*n (%)*]**	1 (1.4)
**HPV prophylactic vaccination [*n (%)*]**	0 (0.0)
**Previous anal examination [*n (%)*]**	0 (0.0)
**Previous anal HPV testing [*n (%)*]**	0 (0.0)
**HPV DNA detection and genotypes [*n (%)95% CI*]**	
Any HPV DNA*	44 (62.8) [51.5-74.2]**
Multiple types of any HPV	33 (47.1) [35.4-58.8]
LR-HPV***	4 (5.7) [0.3-11.2]
HR-HPV****	38 (54.3) [42.6-66.0]
Multiple types of HR-HPV	13 (18.6) [9.5-27.7]
PO-HPV*****	39 (55.7) [44.1-67.4]
HPV-16	0 (0.0) [0.0-4.3]
HPV-18	4 (5.7) [0.3-11.2]
Any 4-valent vaccine types******	7 (10.0) [3.0-17.0]
Multiple 4-valent vaccine types	1 (1.4) [0.0-4.2]
Any 9-valent vaccine types*******	32 (45.7) [34.0-57.4]
Multiple 9-valent vaccine types	7 (10.0) [3.0-17.0]
Non-vaccine HR-HPV types	18 (25.7) [15.5-36.0]

* Any HPV include the following 21 HPV genotypes (HPV-6, −11, −16, −18, −26, −31, −33, −35, −39, −45, −51, −52, −53, −56, −58, −59, −66, −68, −73, −81, and −82).

** The 95% confidence interval (CI) are presented in brackets.

*** LR-HPV include the following 3 LR-HPV genotypes (HPV-6, −11, and −81).

**** HR-HPV include the following 13 HR-HPV genotypes (HPV-16, −18, −31, −33, −35, −39, −45, −51, −52, −56, −58, −59, and – 68).

***** PO-HPV include HPV-26, −53, −66, −73 and −82; LR-HPV include HPV-6, −11 and −81.

****** The 4-valent Gardasil-4^®^ vaccine (Merck & Co. Inc., New Jersey, USA) is effective against HPV genotypes −6, −11, −16 and −18.

******* The 9-valent Gardasil-9^®^ vaccine (Merck & Co. Inc.) is effective against HPV genotypes −6, −11, −16, −18, −31, −33, −45, −52 and −58.

*Nota bene*: HPV DNA detection and genotypes were from all the 70 study MSM.

CI: confidence interval; HPV: Human papillomavirus; HR-HPV: High risk-human papillomavirus; Low risk-HPV: LR-HPV; n: Number; PO-HPV: Possibly oncogenic-HPV.

Generally, the study MSM began their sexual activity around 16 years (15.9±2.1 years), with large variations, ranging from 9 to 22 years. The multipartership was observed in the majority (63.0%) of participants. During the last 6 months, the number of sexual partners was particularly high in the participants, ranging from 1 to 14 (mean number: 6) and they mostly used condom only occasionally (80.0%). The participants reported frequent condomless receptive anal intercourse (82.8%) and receptive oral sex (58.6%), and also less frequent insertive anal intercourse (40.0%) and anal penetration with shared object (20.0%).

Using the national algorithm for HIV testing, only 4 study MSM (5.7%; 95% CI: 0.3–11.2) were found to be infected by HIV. Finally, the study MSM were generally free of clinical STIs symptoms at admission. Thus, only 13 (18.6) participants showed herpetic genital recurrences (n = 5), herpetic anal recurrences (n = 1), syphilis genital ulcer (n = 1), and anal warts (n = 6). No participants had received HPV vaccine or undergone anal cancer screening, clinical anal examination or HPV molecular testing.

**Prevalences of HPV detection and genotypes distribution.** All anal secretions from veil specimens were positive for the ubiquitous TOP3 gene, used as internal control of the BMRT Human Papillomavirus Genotyping Real Time PCR assay. All positive controls had cycle threshold values within the expected ranges. All negative controls were negative without any contamination.

**Fig 2:**
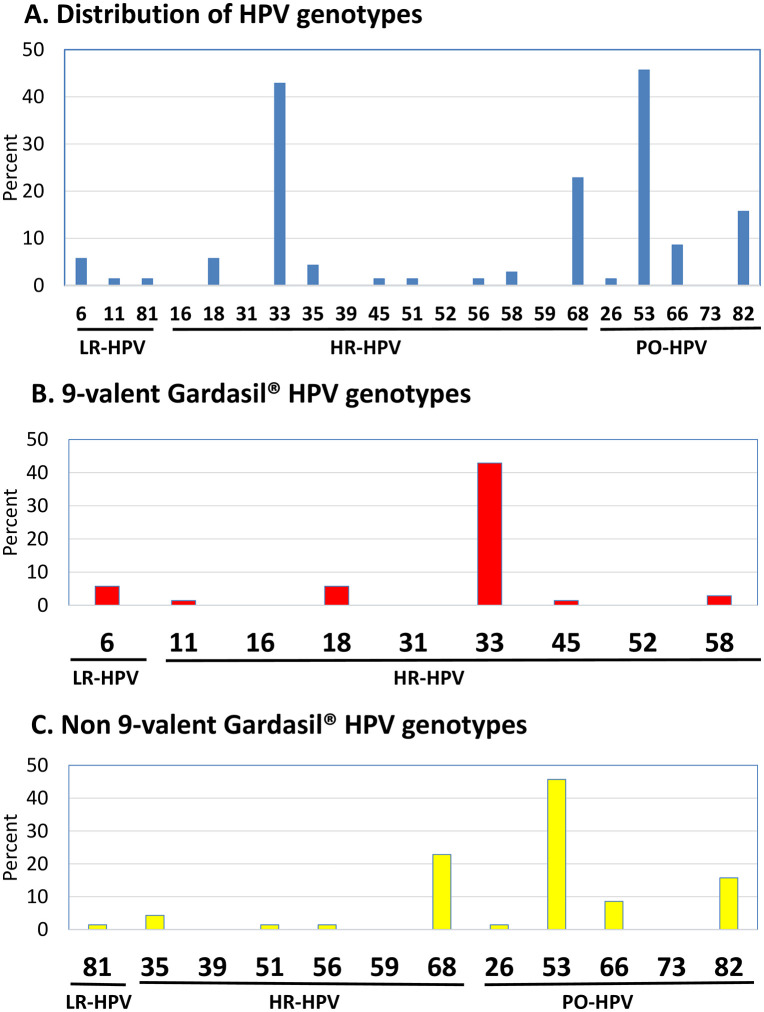
Distribution of HPV genotypes in the study MSM population (N = 70).

The Table 1 and the Fig 2 show the prevalences and distribution of HPV genotypes for any HPV genotypes, HR-, PO- and LR- genotypes and HPV genotypes targeted by the 4- or 9- valent Gardasil-9^®^ vaccines.

Of the veil-based anal self-collected specimens included in the analysis, the majority of MSM participants (n=44) showed anal shedding of HPV DNA, that represented an overall HPV detection prevalence of 62.8%.

The whole distribution of HPV genotypes in HPV-DNA positive anal samples collected by veil is detailed in the Fig 2A. HR-HPV genotypes were frequently detected (38/70; 54.3%). The distribution of genotypes in HR-HPV DNA-positive MSM revealed that HPV-33 (30/70; 42.9%) was the predominant genotype, followed by the HPV-68 (16/70; 22.9%), HPV-18 (4/70; 5.7%), HPV-35 (3/70; 4.3%), HPV-58 (2/70; 2.9%), and HPV-45, HPV-51 and HPV-56 (each type, 1/70;1.4%). PO-HPV were frequently detected (39/70; 55.7%), including HPV-53 (32/70; 45.7%), HPV-82 (11/70; 15.7%), HPV-66 (6/70; 8.6%) and HPV-26 (1/70; 1.4%). LR-HPV were rarely detected (6/70; 8.6%), including HPV-6 (4/70; 5.7%), and HPV-11 and HPV-81 (1/70; 1.4%).

Multiple anal HPV, ranging from 2 to 8 HPV, were frequently observed in 33 (47.1%) MSM. The association of the 3 most frequent HPV, HPV-53+HPV-33+HPV-68, was found in 8 (11.4%) MSM; the association HPV-53+HPV-33 was found in 13 (18.6%) MSM and the association HPV-53+HPV-68 in 6 (8.9%) MSM, and finally the association HPV-33+HPV-68 in 1 (1.4%) MSM. The combination HPV-33+HPV-53 was observed in 21 (30.0%) MSM.

The Gardasil-9^®^ vaccine HR-HPV type 33 and 18 were the predominant HR-HPV genotypes, followed by HPV-58 and HPV-45, and the Gardasil-9^®^ vaccine LR-HPV were HPV-6 and HPV-11 (Fig 2B).

Among all HPV detected, only 9 out the 114 HPV (7.9%), including HPV-18, HPV-6 and HPV-11, were covered by Gardasil-4^®^ vaccine, and 42 HPV (42/114, 36.8%) by Gardasil-9^®^ vaccine, including the HR-HPV-33, -18, -58 and -45, and the LR- HPV-6 and -11. Only 10% (7/70) and 45.7% (32/70) of study MSM were shedding anal HPV covered by Gardasil-4^®^ or Gardasil-9^®^ vaccine, respectively.

The majority of detected HPV were non-covered by Gardasil-4^®^ vaccine (105/114; 92.1%) or by Gardasil-9^®^ vaccine (72/114; 63.1%) (Fig 2C). One-quarter (25.8%) of study MSM were shedding anal non-vaccine HR-HPV.

**Acceptability of anal self-sampling using the Collector V-Veil UP2™ Device.** When asked to choose one collection method, the vast majority (72/75, 96.0%) of study MSM responded that they would prefer the anal self-collection method, demonstrating high acceptability of the anal sampling using the Collector V-Veil UP2™ device of 95.9%. The Table 2 depicts the mean notes given by the participants to the 8 items used to measure the acceptability of veil-based anal self-sampling. Overall acceptability of the anal veil was high, with a note of 3.5 on the Likert scale of acceptability. Three other acceptability items with a high notes (>3) included feeling of high privacy handled during self-collection (mean note of 3.1), low embarrassment during anal self-sampling (mean note of 3.3) and feeling well taken care of by anal self-testing (mean note of 3.3). Two acceptability items showed low notes (<2), including fear of bleeding and feelings of shame during anal self-sampling. All in all, these field observations show a high acceptability of anal self-care with the veil, with a feeling of better care, better intimacy, with little shame or embarrassment, while reporting a limited fear of anal discomfort, anal pain and risk of bleeding.

**Table 2:** Acceptability of the self-collection method with veil (Veil Collector V-Veil UP2™, V-Veil-Up Production SRL) among the 70 study men who have sex with men (MSM) living in N’Djamena, Chad.

Acceptability items*	Veil-based self-collection [mean (SD)]	95%CI
**Overall acceptability**	3.5 (0.6)	[3.4-3.7]
**How well will be your privacy handled during anal sampling?**	3.1 (0.5)	[2.9-3.2]
**Will you feel embarrassed during anal sampling?**	3.3 (1.2)	[3.0-3.6]
**Will sampling cause you any anal discomfort?**	2.4 (1.5)	[2.0-2.8]
**Will sampling cause you any anal pain?**	2.9 (1.7)	[2.5-3.3]
**Are you afraid of bleeding during anal self-sampling?**	1.9 (0.8)	[1.7-2.1]
**How shameful are you when anal sampling?**	1.4 (0.5)	[1.3-1.5]
**How well cared for will you feel because anal sampling?**	3.1 (0.8)	[2.9-3.3]

* The scale of acceptability was assessed by a Likert scale ranging from 1 (most difficult) to 4 (= most favorable); the results are mean ±1 standard deviation (SD); 95% confidence intervals (CI) are given in brackets.

**Usability and satisfaction of anal self-sampling using the Collector V-Veil UP2™ device.** The results of the face-to-face questionnaire for usability and satisfaction regarding veil-based self-sampling using the Collector V-Veil UP2™ device are depicted in the Table 3, showing the mean notes given by the participants to the 10 items used to measure the usability of veil-based anal self-sampling, mainly focused on general understanding of the instructions for use (4 items) and of the component’s device (3 items) and the practical use of the anal veil (3 items), and to the 11 items of the satisfaction questionnaire.

**Table 3:** Usability and satisfaction of the self-collection method with veil (Veil Collector V-Veil UP2™, V-Veil-Up Production SRL) among the 70 study men who have sex with men (MSM) living in N’Djamena, Chad.

Variables*	Veil-based self-collection [mean (SD)]	95%CI
**Understanding of instruction for use**		
General understanding	3.0 (1.1)	[2.5-3.1]
Instructions for use	2.9 (1.2)	[2.6-3.2]
Verbal explanations of instructions for use	3.7 (0.5)	[3.6-3.8]
Anatomic sketches	3.1 (0.6)	[2.9-3.2]
**Understanding of component’s device**		
Device has two components (veil; applicator)	3.7 (0.5)	[3.6-3.8]
After collecting anal secretions, the veil must be placed in a pot	3.7 (0.4)	[3.6-3.7]
Your study code must be written on the label	3.2 (0.6)	[3.0-3.3]
**Practical use of anal veil**		
Applicator to be placed in the pocket	3.7 (0.6)	[3.5-3.8]
Place the veil deep (2–3 cm) in the anal canal and rotate it twice	3.3 (0.7)	[3.1-3.4]
A period of at least 4 hours after defecation must be respected	3.4 (0.8)	[3.2-3.6]
**General satisfaction**		
Overall feeling about this anal self-collection?	3.8 (0.8)	[3.6-3.9]
The veil allows to respect your intimacy	3.3 (0.6)	[3.1-3.4]
Was it easy to collect canal anal secretions with self-collection?	1.9 (1.1)	[1.6-2.1]
How ashamed were you to perform this anal self-collection?	3.1 (0.9)	[2.9-3.3]
How confident are you that you used the veil correctly?	2.1 (0.8)	[1.9-2.3]
Did you experience any discomfort during veil-based self-sampling?	2.0 (0.8)	[1.8-2.2]
Did you experience any pain during veil-based self-sampling?	1.1 (0.8)	[0.9-1.3]
Did you experience bleeding during veil-based self-sampling?	1.0 (0.6)	[0.8-1.1]
Did you experience itching during veil-based self-sampling?	1.0 (0.3)	[0.9-1.1]
Do you prefer to make the veil-based self-sampling at hospital?	1.0 (0.2)	[0.9-1.0]
Do you prefer to make the veil-based self-sampling at house?	3.7 (0.5)	[3.6-3.8]

* The scale of satisfaction was asses by a Likert scale ranging from 1 (= less favorable) to 4 (= most favorable); the results are mean ± 1 standard deviation (SD); 95% confidence intervals (CI) are given in brackets.

Most participant MSM (65; 92.8%) reported that the instructions for use written in French were easy to read and to understand, while the verbal explanations on how to use the collection device showed high mean note according to the Likert scale of satisfaction (mean note for oral explanation: 3.7/4). The large majority of participant MSM (68; 97.1%) were able to recognize correctly the component’s device, with high notes (mean note of 3.7), to understand that the impregnated veil must be placed in dedicated pot after sampling (mean note of 3.7), with clear pre-analytical identification (mean note of 3.2). The practical items for usability gave high notes for each item including correct placement in the anal canal (mean note of 3.3), and respect of waiting period of at least 4 hours after defecation (mean note of 3.4).

Mean notes concerning the general satisfaction of the Collector V-Veil UP2™ device showed mean notes from 1.0 to 3.0. The low notes concerned rarely anal pain, bleeding or itching, and anal discomfort. The high notes (>3.0) were associated with high overall feeling (mean note of 3.8), intimacy respect (mean note of 3.3), and lack of shame (mean note of 3.1). When asked to choose one collection method, 67 (95.7%) of study participants responded that they would prefer the self-collection method. Furthermore, most participants (64; 91.5%) reported that they would be willing to perform veil-based anal collection at home and bring the specimen with them to clinic (mean note of 3.7), rather than to hospital or other medical facilities (mean note of 1.0).

## Discussion

Little is known about anogenital HPV carriage in MSM in Central Africa. We conducted the first evaluation in Africa, to our knowledge, of the acceptability, usability, and satisfaction of veil-based anal self-sampling among young, low-education MSM in N’Djamena, Chad. Additionally, we assessed anal shedding of HPV genotypes to predict the potential efficiency of prophylactic HPV vaccines. All anal specimens collected with the veil were positive for the ubiquitous TOP3 gene, confirming adequate cellular DNA for molecular testing. Our study yielded four main findings. First, HIV seroprevalence among participants reached 5.7%, about six times higher than in the general adult population in Chad, confirming MSM as a key population for HIV and STIs in a generalized epidemic. Second, the Collector V-Veil UP2™ device showed high acceptability and usability. Participants found the instructions clear, reported correct placement in the anal canal, and expressed high satisfaction, highlighting comfort, privacy, and absence of shame. Third, the distribution of HPV genotypes was atypical, showing a mix of HR-HPV, PO-HPV, and LR-HPV, with HPV-33 and HPV-68 predominating among HR-HPV, HPV-53 and HPV-82 among PO-HPV, and HPV-6 among LR-HPV. This pattern likely reflects selection bias introduced by RDS recruitment, which may have captured participants within the same sexual network. Fourth, most detected HPV genotypes (63.1%) were not covered by Gardasil-9^®^, and one-quarter of participants harbored non-vaccine HR-HPV, suggesting limited vaccine efficacy in this population. Taken together, these results highlight MSM in N’Djamena as a specific core group for HIV and HPV transmission. Although HIV prevalence was higher than in the general population, it affected only one in twenty MSM. The RDS method likely recruited individuals from the same sexual network, producing a unique HPV profile and limited predicted vaccine coverage. These findings contrast with the genital HPV distribution in young women in N’Djamena, who commonly harbor preventable Gardasil-9^®^ genotypes [26,41]. Given the potential recruitment bias, our observations may not be generalizable to all MSM in Chad. A larger survey, similar to the Integrated Biological and Behavioral Surveillance Survey (IBBSS) [42], is recommended.

MSM in Chad face barriers to HIV services due to limited specialized care and pervasive stigma. The HIV seroprevalence of 5.7% in our cohort aligns with national estimates for MSM reported by the Global Fund (4.8%) [43] and UNAIDS (3.9–9.0%) [25,44]. Rates remain far lower than those observed in neighboring Central African countries: the Central African Republic (41–69%) [9,20], Cameroon (12.9–44.4%) [6,45,46], and Nigeria (25%) [47]. In Western and Central Africa, a systematic review and a meta-analysis reported high HIV prevalence reaching 25.1% among MSM, with an 11.3-fold higher risk than in the general population [46,48]. The relatively lower prevalence in Chad suggests a more contained epidemic, though confirmation with a larger, less biased sample is needed. Currently, there is no effective national “minimum package of services” for MSM offering HIV screening, pre-exposure prophylaxis (PrEP), and medical care [49]. Tailored prevention and treatment interventions are urgently required to achieve Chad’s zero-incidence target by 2030 [50].

Feasibility of veil-based anal self-sampling included operational (acceptability, usability) and biological (technical) aspects. Operationally, self-sampling was highly practical and well accepted (96.0%), with usability rated 92.3%. Most participants found instructions easy to understand (92.8%), correctly positioned the device (mean 3.3/4), and understood verbal explanations (3.7/4). The process was painless, easy, and respected privacy. A large majority (91.5%) would be willing to perform self-sampling at home and deliver samples to a clinic, supporting feasibility for broader screening. Biologically, all veil specimens were positive for the TOP3 gene, confirming DNA adequacy for HPV detection by real-time PCR. The veil design allows full recovery of biological material, and environmental conditions such as temperature or transport time do not compromise sample quality.

Self-collection proved highly acceptable, comparable to rates (90–98%) reported among MSM in North America [51–56]. In the UK, acceptability ranged from 62.5–84% [57,58], and in France, from 91–97% among HIV-infected populations [59,60]. In the USA, rates varied between 63–78% among HIV-positive and -negative women [61,62]. High acceptability is consistent across studies [63], though influenced by recruitment venue and on-site collection [58,64]. Our participants appreciated privacy and low embarrassment, as seen previously [52,61]. Fear of pain or bleeding was minimal, and the device’s illustrated instructions improved usability even among low-education participants. Cultural adaptation of self-sampling tools enhances feasibility across groups [65]. Participants emphasized privacy and respect for intimacy, consistent with prior findings that home-based sampling increases screening uptake [55]. As with self-collected cervical samples for HPV screening [66–69], anal self-sampling can extend access to populations outside traditional healthcare systems [60]. The Collector V-Veil UP2™ device was painless, hygienic, and easy to use. Self-collection methods are valid for STI and HPV testing in men [28,29,70], including for cytology in anal intraepithelial neoplasia detection [71]. Implementing anal self-sampling could expand HPV detection and treatment of HR-HPV lesions [72]. Environmental conditions generally do not affect sample adequacy except for fecal contamination [73]. Offering self-collection options based on MSM preferences is likely to increase screening coverage, and our results confirm the veil’s suitability.

The prevalence of anal HPV (~63%) was high among MSM in N’Djamena. Anal HR-HPV prevalence (~54%) aligns with findings from Central and West Africa—Central African Republic, Liberia, Mali, Nigeria, Rwanda, South Africa, and Togo—where rates range from 21% to 72% [9,15,19,23,74–77], though lower than 87% in young Black American MSM [78]. Studies elsewhere reported 29–56% [79–83]. Anal HPV thus represents a significant public health issue among MSM in Chad, emphasizing the need for targeted prevention strategies.

The HPV genotype distribution in this study was distinctive, with high rates of PO-HPV and HR-HPV. The most frequent were PO-HPV-53 (45.7%), HR-HPV-33 (42.9%), and HR-HPV-68 (22.9%). This contrasts sharply with Chadian women, in whom HPV-58, HPV-35, HPV-56, HPV-31, HPV-16, HPV-45, HPV-52, and HPV-18 predominate [26]. However, similar patterns of HPV-53 and HPV-68 dominance were seen in women from the Democratic Republic of Congo [84,85], suggesting possible co-circulation of these genotypes regionally. Multiple HPV infections were common, observed in nearly half the cases; HPV-33 + HPV-53 appeared in one-third, and HPV-33 + HPV-53 + HPV-68 in one in ten MSM. HR-HPV-18 appeared in ~5%, and HR-HPV-16 was absent. This profile differs markedly from neighboring Central African Republic and Nigeria. In the Central African Republic, HPV-35 predominated, followed by HPV-58, HPV-59, and HPV-31, with multiple infections similar to ours [9,22]. Nigerian MSM primarily harbored HPV-16, HPV-45, HPV-51, HPV-35, and HPV-18, with multiple infections in 80% [86]; another study found HPV-35, HPV-58, and HPV-51 more frequent than HPV-18 or HPV-16 in HIV-positive MSM [19]. In Rwanda, HPV-16 was the most common, followed by HPV-18, HPV-35, and HPV-53 [77]. No data yet exist for MSM in Cameroon, Congo, or Gabon. Regarding LR-HPV, HPV-6 predominated as elsewhere in SSA [2,3,86]. Overall, the HR-HPV distribution among MSM in N’Djamena appears unique, featuring genotypes uncommon in Western or Southern African contexts [22,86,87].

The RDS recruitment method probably introduced a selection bias, capturing MSM from the same sexual network, thus concentrating certain HPV strains. RDS is widely used to reach hidden populations such as MSM [32,88,89], but its reliance on peer recruitment can overrepresent specific social clusters [32,88]. These findings point to regional diversity in HPV molecular epidemiology among MSM in sub-Saharan Africa [24]. It is plausible that anal cancers in some African MSM populations may be driven by HR-HPV genotypes other than HPV-16 and HPV-18, which predominate in Western countries [87,90].

Most HPV genotypes detected (63.1%) were not covered by Gardasil-9^®^, and one in four MSM shed non-vaccine HR-HPV (25.8%), suggesting limited potential vaccine efficacy. This contrasts with the 70% predicted coverage in Chadian women [26]. In other African MSM cohorts, vaccine coverage predictions were higher: Nigeria (51.5%) [86], Mali (54%) [15], South Africa (57%) [23], and the Central African Republic (68.9%) [9]. Thus, Gardasil-9^®^ could prevent most HR-HPV infections in many African MSM groups, but not all. Non-vaccine HR-HPV infections remain common, as seen in the Central African Republic and Mali [9,15]. Hence, current vaccine formulations may inadequately protect significant MSM subpopulations. The HPV immunization guidelines of the US Advisory Committee on Immunization Practices [91,92], which recommend Gardasil-9^®^ for MSM up to 26 years old, may need adaptation for sub-Saharan African contexts.

Our study had several limitations. Although the combination of RDS and self-sampling with the Veil Collector V-Veil Up UP2™ device offers an innovative approach to engage MSM in low-resource and stigmatized contexts, it inherently compounds risks of selection bias and misclassification. Specifically, it may produce an atypical HPV genotype profile that reflects dense sexual network clustering rather than true prevalence, moreover considering the relatively low number of patients included. Thus, the representativeness of the included study population is not ensured. Indeed, the recruitment of participants using the RDS method by a single NGO in N’Djamena focused on the care of key populations may have introduced several biases, including recruitment bias in which initial seeds may not be representative of the target population, referral bias, in which participants may preferentially refer others similar to themselves, leading to oversampling of certain subpopulations, and network effects involving the social network structure which can influence recruitment patterns, potentially distorting the sample. The possible recruitment through NGOs may have favored individuals already linked to care. The observed genotype distribution may reflect network effects more than true population-level prevalence. Furthermore, the sample size of our study population was small accentuating the risk of major selection bias. The small sample size may be attributed to the inherent difficulty of recruiting MSM in African context, the short 2-months duration of recruitment, and the rainy season, which is very intense in Chad in August and September, resulting in numerous logistical difficulties. Thus, the study participants may be not completely representative of the MSM community of Chad, especially regarding the prevalence of HIV and anal HPV infection, as well as the distribution of HPV genotypes. In addition, participants were included on a voluntary basis, that may constitute another source of recruitment bias, impacting the validity of the answers to sociodemographic questionnaire, including items related to the intimacy of their life. The face-to face questionnaire carried out by health care observers could have introduce a bias influencing response quality and accuracy. Otherwise, we were not aware of true disease status of study participants, since we did not perform any pathological evaluation, including anal cytology or anoscopy. Furthermore, our results on the acceptability, usability and satisfaction of anal self-sampling using the Collector V-Veil UP2™ device in the Chad MSM population are new, original, and have never been reported in any study before, to the best of our knowledge, making it impossible to compare or discuss them in other contexts. Another limitation of the study is the lack of direct diagnostic validation of the anal veil-based self-collection device against a clinical gold standard that limits confidence in the sensitivity of the method, although two previously published studies comparing the efficiency of veil-based self-sampling to classical provider-based collection of genital secretions showed that vaginal specimens self-collected through the veil provided better detection of oncogenic HPV than collection by swab [41] or scraping [93]. Thus, the interpretation of negative results and the estimation of the actual vaccine-preventable burden of HPV in the MSM population should be done with caution. All in all, unvalidated anal self-sampling without a comparison sample using clinician-based anal swabbing could represent a bias. Finally, we did not carry out multivariate analyses with study analytical data. Indeed, the validity of multivariate analysis in RDS population with the aims to reach hidden and hard-to-reach MSM population in N’Djamena was intrinsically too much complex, with a breakdown of the key analysis considerations, including mainly sampling biases, lack of control of network effects and network-level variables (*e.g.,* recruitment depth) as covariates in multivariate models, and multiple waves of recruitment mitigating the impact of initial seed selection bias.

## Conclusion

In conclusion, the MSM community living in N’Djamena constitutes a vulnerable population at high-risk for both HIV and HR-HPV anal infections. MSM in Chad should urgently receive adapted STIs and anal cancer prevention, screening and care, with the implementation of innovative and convenient preventive interventions against HPV infection and associated cancers.
